# 1177. Mind the GAPP: Global Arrivals and Pre-treated Patients screening at a children’s cancer center

**DOI:** 10.1093/ofid/ofac492.1014

**Published:** 2022-12-15

**Authors:** Sheena Mukkada, Anita Max, Tessa Denzin, Ghussai Abd El Gadir, Elisabeth Adderson, Hana Hakim, Randall Hayden, Gabriela Maron, Joshua Wolf

**Affiliations:** St. Jude Children's Research Hospital, Memphis, Tennessee; St. Jude Children's Research Hospital, Memphis, Tennessee; St. Jude Children's Research Hospital, Memphis, Tennessee; Erlanger Health System, Chattanooga, Tennessee; St. Jude Children's Research Hospital, Memphis, Tennessee; St. Jude Children's Research Hospital, Memphis, Tennessee; St. Jude Children's Research Hospital, Memphis, Tennessee; St. Jude Children's Research Hospital, Memphis, Tennessee; St. Jude Children's Research Hospital, Memphis, Tennessee

## Abstract

**Background:**

Clinical and laboratory evaluation for infectious diseases prior to initiation of cancer-directed therapy creates opportunities for risk assessment, infection prevention and targeted treatment. The need may be greater for patients from outside the United States (US) where the epidemiology of infectious diseases may differ. We created the Global Arrivals and Pretreated Patients (GAPP) program to address this need at a referral hospital for children with cancer and blood disorders.

**Methods:**

Patients accepted for treatment at our hospital are referred to GAPP for the following reasons: 1) residence in the continental US for 1 year or less, 2) travel outside the continental US within 3 months of referral 3) hospitalization outside the continental US, or 4) clinician request. GAPP evaluation includes review of history and laboratory studies, and recommendations for screening tests and management.

**Results:**

Between March 21, 2019 and May 2, 2022, 35 patients were evaluated; 9 (26%) with solid or CNS tumors, 25 (71%) with hematological malignancies and 1 (3%) with immune thrombocytopenic purpura. Of these, 20/35 (57%) were from Latin America, and 10/35 (29%) from Eastern Europe. Most patients (29/35 [83%]) were seen within 2 weeks of arrival to the institution. Evaluation prompted isolation precautions in 2/35 (6%) for communicable respiratory illness and in 4/35 (11%) for ESBL-producing organism on perianal screen. Additionally, 13 patients were under-immunized (mostly non-immune to hepatitis B [7/35, 20%] or varicella zoster virus [8/35, 22%]), and 9/35 (26%) had an exposure-related risk. Finally, parasitic infection prompted specific management in 4 patients; 3/35 (9%) had positive serology for *Toxoplasma gondii* requiring appropriate prophylaxis, and 1/35 (3%) was treated for cryptosporidiosis.

**Conclusion:**

Screening new arrivals to the US prior to administration of immunosuppressive therapy is feasible and may identify risk factors for illness during therapy as well as opportunities for infection prevention, immunization, and treatment or suppression of existing infections. Providers focusing on acute hematologic or oncologic illness may otherwise miss this quality improvement opportunity in a patient group at high risk of poor infectious outcomes.

**Disclosures:**

**Randall Hayden, MD**, Inflammatix: Advisor/Consultant|Quidel Corporation: Advisor/Consultant|Roche Diagnostics: Advisor/Consultant|T2 Diagnostics: Advisor/Consultant **Gabriela Maron, MD**, Astellas Inc,: Grant/Research Support|SymBio Pharmaceuticals: Grant/Research Support **Joshua Wolf, MBBS, PhD**, Karius: Grant/Research Support|Merck: Participation in industry sponsored research.

